# Manual of Tropical Medicine

**Published:** 1911-03

**Authors:** 


### Reviews of Books.

**Manual of Tropical Medicine.** By ALDO CASTELLANI, M.D., and ALBERT J. CHALMERS, M.D. Pp. xxv, 1242. London: Baillière, Tindall & Cox. 1910.

We have been accustomed to a high standard in text-books of tropical medicine by the writings of Manson, Daniels, and others, but the present volume worthily keeps up the tradition. Its authors are thoroughly qualified for their work. Castellani is Director of the Clinique for Tropical Diseases in Colombo, and also served on the Sleeping Sickness Commission in Uganda. To him we owe the discovery of the trypanosome in sleeping sickness, and of the spirochæta pertenuis of yaws. Chalmers is pathologist and parasitologist at Colombo, and has also seen service in West Africa.

To a large extent the ground covered is similar to that in Manson's well-known book, and the descriptions are clear and interesting. An immense space is of course taken up by cataloguing and differentiating the multitude of Biting Flies, Ticks, Fleas, Worms, Protozoa, and other parasites, in which a beginner would rather lose his way, but which to one who had taken out a course in Tropical Medicine would be invaluable. The illustrations are numerous and excellent.

Distinctive features of the book are the accounts of Indian and other native poisons, an admirable chapter on snakes, and especially a full and detailed description of the various tropical skin diseases which so often puzzle the doctor. On this subject Castellani is peculiarly at home.

He is quite confident that yaws and syphilis are distinct diseases, caused by closely allied spirochætes. Man or monkeys immune to the one can readily be inoculated with the other.

Needless to say, the book is thoroughly up to date, including even an account of cholera carriers of the type rendered familiar to us by the researches of Drs. Davies and Walker Hall on typhoid.

Space is saved by omitting all reference to laboratory methods, for which the authors refer to other special text-books, but the directions for treatment are full and minute.

As a matter of taste, we must enter a protest against such an innovation in spelling as agchylostomiasis.

It is safe to prophesy that most medical practitioners in isolated situations in the tropics will now feel it necessary to take this book as well as others. Men reading for the higher medical examinations in England, or having tropical cases under their care, will also do well to consult such an excellent manual.

